# Hepatic endometriosis: a rare case and review of the literature

**DOI:** 10.1186/s40001-015-0137-1

**Published:** 2015-04-04

**Authors:** Kai Liu, Wei Zhang, Songyang Liu, Bingfei Dong, Yahui Liu

**Affiliations:** Department of Hepatobiliary and Pancreatic Surgery, No.71 Xinmin Street, First Hospital, Jilin University, Changchun, 130021 China; Department of Thyroid Surgery, No.71 Xinmin Street, First Hospital, Jilin University, Changchun, 130021 China

**Keywords:** Hepatic endometriosis, Hepatic cysts, Atypical endometriosis, Differential diagnosis

## Abstract

Hepatic endometriosis is one of the rarest disorders characterized by the presence of ectopic endometrium in the liver. To our knowledge, only 21 cases of hepatic endometrioma have been described in the medical literature. We report a case of a 36-year-old primiparous female with hepatic endometriosis forming a large cystic mass. The patient presented once with severe right quadrant pain as her only symptom and no history of endometriosis. Complete blood count and biochemical tests were normal. Abdominal ultrasonography and computed tomography scans suggested the presence of a 6.5 × 6.0 cm cystic mass in segment III of the liver. The mass was completely removed by local liver resection. The intraoperative frozen sections suggested a diagnosis of hepatic endometriosis. The diagnosis was confirmed through histological immunostaining without intrinsic abnormality. A preoperative diagnosis of hepatic endometriosis is made on the basis of considering the possibility in advance. Hepatic endometriosis should be considered in the differential diagnosis of a cystic liver mass despite conducting exhaustive investigations in the absence of characteristic clinical and radiological features. Histological examination is essential, and surgery remains the treatment of choice.

## Background

Endometriosis is a benign condition most commonly noted in the uterus, fallopian tubes, ovaries, and local pelvic peritoneum [1]. It affects approximately 10% of women of reproductive age and 2.5% of postmenopausal women [2]. However, endometriotic lesions have also been described in almost all other remote organs of the human body, including the omentum, gastrointestinal tract, peritoneum, operative scars, lymph nodes, umbilicus, skin, lungs, pleura, bladder, kidneys, pancreas, and even in males [3]. Hepatic endometriosis, one of the rarest forms of atypical endometriosis, was first described in 1986 [4]. To our knowledge, only 21 cases of hepatic endometriosis have been previously reported in the literature. We herein describe the 22nd case of hepatic endometriosis and evaluate the current literature addressing the diagnosis of hepatic endometriosis focusing on advances in the clinical manifestation, pathogenesis, and diagnostic workup.

## Case presentation

**Figure 1:**
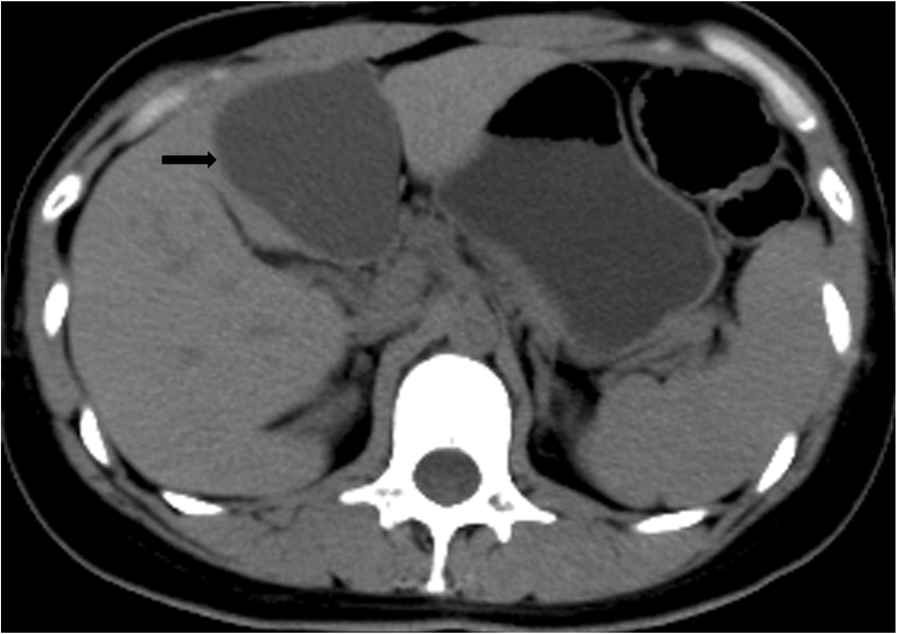
**Hepatic CT scan showing a well circumscribed cystic lesion of 6.5 × 6.0 cm located in segments III of the left liver lobe.**

A 36-year-old woman, primigravida, consulted our hospital for investigation of a cystic hepatic lesion in August 2013. The patient had no history of endometriosis. Half a year previously, the patient had presented with severe right quadrant pain in the midnight which only lasted for 3 h just before menstruation. An upper abdominal ultrasound showed a 6-cm lesion within the left lobe of the liver. The patient was diagnosed with a hepatic cystic mass in segment III of the left lobe. A laparotomy had been considered and postponed since the patient had managed to cope with her symptoms. The patient refused further treatment at that moment. The patient was not currently undergoing menopause. The patient had no exposure to hepatotoxic drugs, estrogens, progestins, or oral contraceptives. There were no other significant symptoms. On physical examination, no definite abdominal mass lesion was palpable and no lymphadenopathy was noted. Complete blood count was within normal range as were the liver function tests. Serological tests for hepatitis B surface antigen and anti-hepatitis C virus antibodies were negative. The tumor markers (carcino-embryonic antigen, carbohydrate antigen 19-9, alpha-fetal protein, carbohydrate antigen 125) were normal, as was the routine lab work. A preoperative computed tomography scan showed a well-circumscribed cystic lesion of 6.5 × 6.0 cm located in segment III (Figure 1). The wall appeared thick with complex septate. A preoperative tentative diagnosis included liver cystadenoma or liver cystadenocarcinoma.

**Figure 2:**
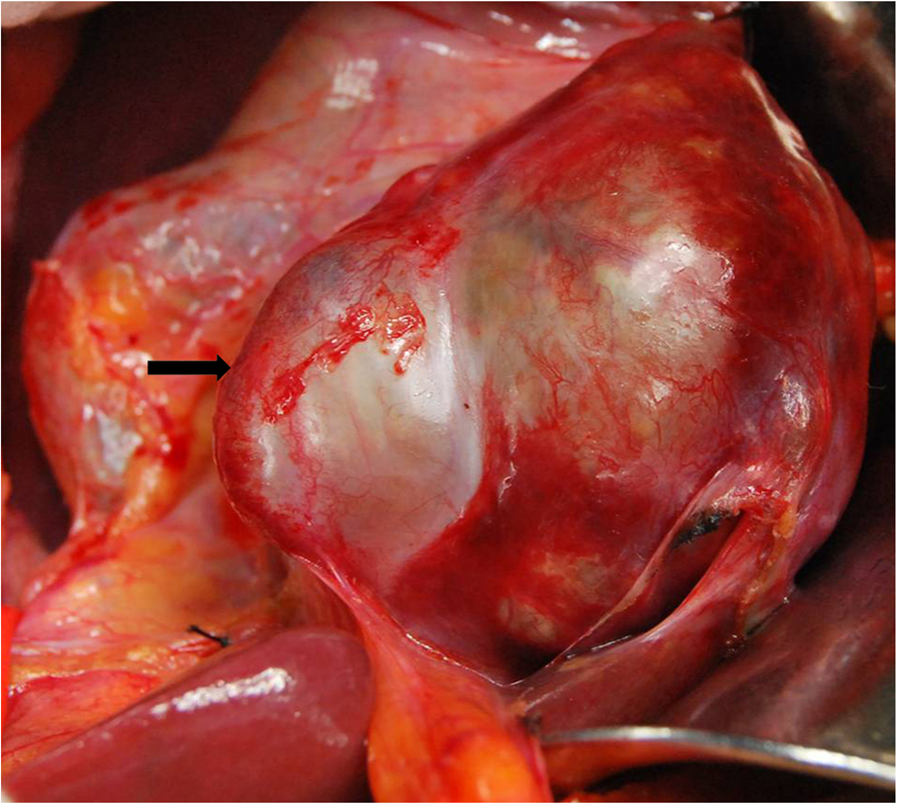
**Macroscopic examination of the liver mass.** A 6.5 × 6.0 × 5.5 cm cystic mass was presented in the left lobe of the liver with thick fibrous capsule.

An exploratory operation was performed, which revealed a large cystic tumor occupying segment III of the liver (Figure 2). There was no evidence of metastases. Biopsies were taken from the cyst wall, and the intraoperative frozen section histology suggested a diagnosis of hepatic endometriosis. The endometriosis was completely removed by a pericystectomy. An abdominal cavity exploration revealed no other pathologic events. The pelvis was examined and no evidence of endometriosis was found. We did not detect any other abnormalities during the operation. The patient made an uneventful recovery and was discharged on the ninth postoperative day. After 3 months of follow-up, the patient is asymptomatic with no evidence of recurrent disease.

**Figure 3:**
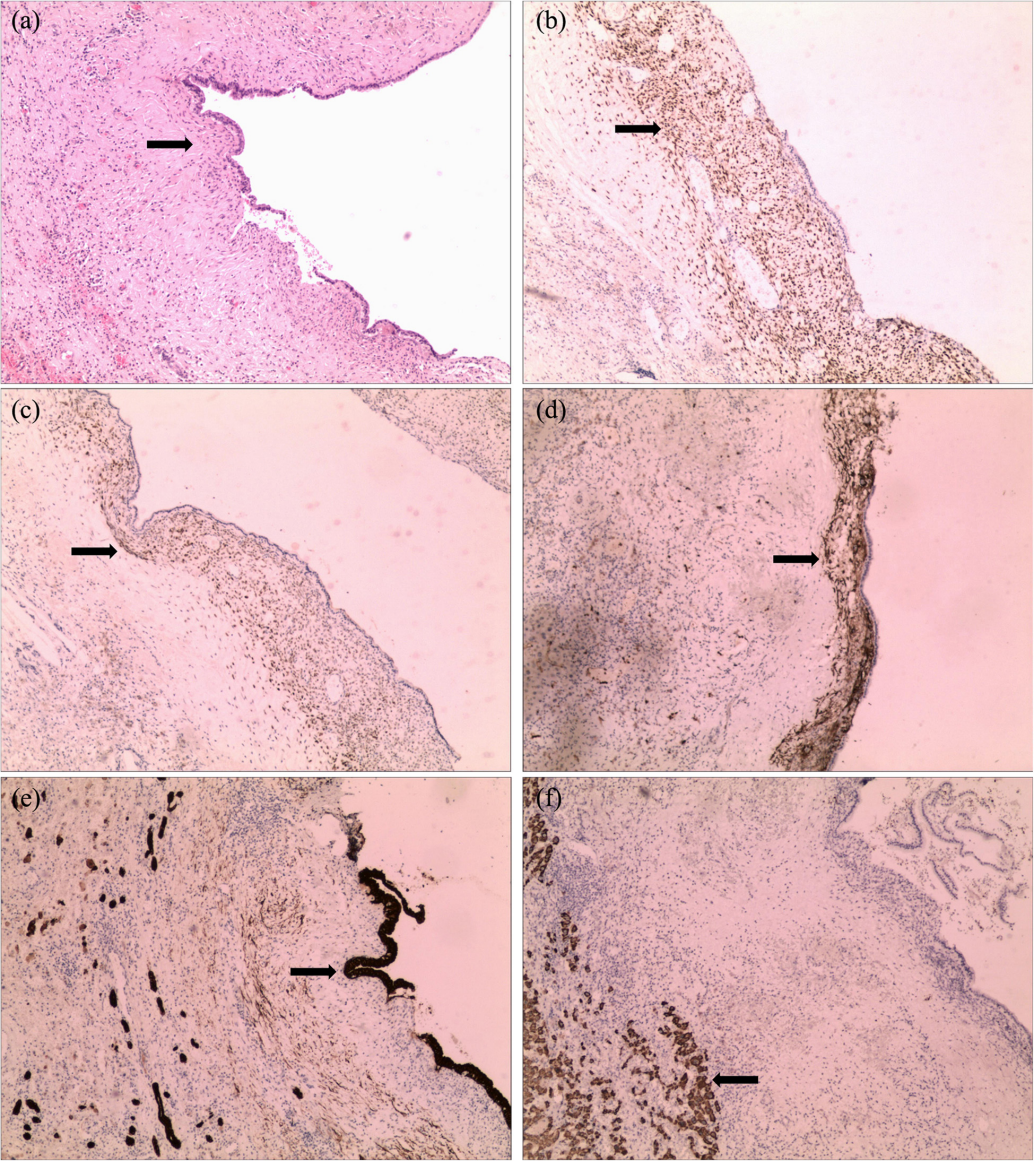
**Microscopic examination of the endometriosis cyst of the liver.** Cyst and normal liver tissue stained with hematoxylin and eosin **(a)** and with immunohistochemistry with estrogen receptor antibody **(b)**, progesterone receptor antibody **(c)**, CD10 antibody **(d)**, CK7 antibody **(e)**, and HepPar-1 antibody **(f)** (×100 magnification). No hyperplasia or atypia was observed in epithelial or stromal component.

Histopathology analysis revealed a lobulated cyst with adjacent normal hepatic parenchyma. The cyst wall was partially composed of endometrial glandular and stromal elements (Figure 3a), characteristic of endometriosis. Immunostaining of the stromal cell and epithelial cells expressed strong coloring for estrogen and progesterone receptor (Figure 3b,c). This was further confirmed by positive immunostaining of CD10, CK7, as well as HepPar-1, which proved the epithelial origin of the cyst (Figure 3d,e,f). Since no atypical cells were detected, we concluded the diagnosis to be benign intrahepatic endometriosis.

## Discussion

Endometriosis is defined as the presence of endometrial-like tissue outside the uterus, which induces a chronic, inflammatory reaction [1]. It affects approximately 10% of women of reproductive age and 2.5% of postmenopausal women, from all ethnic and social groups [2]. Although clinical and imaging features of typical endometriosis within the pelvis can be highly suggestive, the gold standard for diagnosis is based on the histologic examination of appropriately sampled tissue [5]. Endometriosis was first described by Rokitansky in 1860. The most commonly affected sites are the pelvic organs (ovaries, fallopian tubes, uterosacral ligaments, Pouch of Douglas) and peritoneum, although other parts of the body such as the lungs are occasionally affected. However, endometriotic lesions have also been described in some other unusual remote organs of the human body, including the omentum, gastrointestinal tract, operative scars, lymph nodes, umbilicus, skin, pleura, bladder, kidneys, pancreas, and even in males [3, 6].

However, the mechanism by which endometriosis is involved is still largely unknown. Significant effort has been undertaken to identify potential causes in the development of endometriosis, including retrograde menstruation, coelomic metaplasia, iatrogenic injury, and hematogenous or lymphatic dissemination [7]. Retrograde menstruation is believed to cause transcoelomic spread and implantation within the pelvis due to its predilection for gravity-dependent pelvic deposits in most observed lesions. However, this theory cannot explain distant and intraparenchymal lesions in atypical cases while the haematogenous or lymphatic dissemination may offer a better explanation for these atypical locations. The possible transportation of endometrial fragments is based on these vessels as vehicles in a fashion analogous to metastatic spread of neoplasm. The coelomic metaplasia may be, at least in part, due to chronic inflammation, hormonal alternation, or trauma to promote endometriosis and that might be amenable to disruption in the male or heart [6, 8]. However, laboratory findings and clinical data of hepatic endometriosis fell short of metaplasia theory as most of the hepatic lesions are intraparenchymal.

Hepatic endometriosis, first described by Finkel *et al.* in 1986, is an extremely rare disorder characterized by the presence of ectopic endometrial tissue in the liver [4]. The first reported patient was a 21-year-old female who presented with epigastric pain, nausea, and vomiting. The patient was found to have an endometrial cyst measuring 13 cm in diameter located in the left lobe of the liver. Subsequently, 21 cases of this rare form of endometriosis have been described in the medical literature. All cases of previously reported hepatic endometrioma and our present case are presented in Table 1. In our present case, the patient had no history of endometriosis or previous pelvic operation. By profiling the clinical and biological properties of the hepatic lesion, we may therefore identify vascular dissemination whose importance in hepatic intraparenchymal endometriosis was poorly recognized. We have now determined that in our case, the liver maybe a transportation target of endometrial fragments by lymphatic or blood vessels.

**Table 1:** **Feature of reported cases of hepatic endometriosis in literature**

**Reference**	**Age (years)**	**Liver involvement**	**Endometrial history**	**Pre-operation diagnosis**	**Treatment**
Finkel *et al.* [4]	21	Left lobe	No	No	Cyst enucleation
Grabb *et al.* [9]	21	Left lobe	No	No	Deroofing + danazol
Rovati *et al.* [10]	37	Left lobe	Yes	Yes	Segmentectomy + danazol
Verbeke *et al.* [11]	62	Left lobe	No	No	Excision
Verbeke *et al.* [11]	34	Right lobe	No	No	Right hepatectomy
Chung *et al.* [12]	40	Left lobe	Yes	No	Segmentectomy
Weinfeld *et al.* [13]	60	Right lobe	Yes	No	Left hepatectomy + excision
Inal *et al.* [14]	25	Right lobe	Yes	No	Danazol
N’senda *et al.* [15]	54	Right lobe	No	Yes	Right hepatectomy
Huang *et al.* [16]	56	Left lobe	Yes	No	Left hepatectomy
Jeanes *et al.* [17]	31	Bilobar	Yes	Yes	Right hepatectomy
Khan *et al.* [18]	31	Bilobar	Yes	Yes	Right hepatectomy + goserelin
Khan *et al.* [18]	59	Right lobe	Yes	Yes	Right hepatectomy
Tuech *et al.* [19]	42	Right lobe	No	No	Excision
Reid *et al.* [20]	46	Right lobe	Yes	No	Right hepatectomy + goserelin
Groves *et al.* [21]	52	Right lobe	No	No	Right hepatectomy
Goldsmith *et al.* [22]	48	Left lobe	Yes	No	Non anatomical resection
Asran *et al.* [23]	61	Bilobar	Yes	Yes	Unknown
Schuld *et al.* [24]	39	Right lobe	No	No	Segmentectomy
Fluegen *et al.* [25]	32	Right lobe	No	No	Pericystectomy
Rivkine *et al.* [26]	51	Left lobe	No	No	Left hepatectomy
Liu *et al.*, this study	36	Left lobe	No	No	Pericystectomy

The clinical, biological, and morphological characteristics of hepatic endometriosis need to be reported in the literature to allow for the creation of a better diagnostic approach. These reported data suggest that, no specific diagnostic marker may be sufficient to isolate hepatic endometriosis from hepatic lesions except a histological examination, despite conducting a complete investigation, in the absence of clinical and radiological characteristics. Prospective identification of hepatic endometriosis has limitations that contribute to the controversy surrounding their existence. Although transhepatic biopsy for hepatic endometriosis have been sufficient to differentiate other hepatic lesions, it is difficult to make a preoperative diagnosis for the majority of hepatic endometriosis patients due to the potential adverse effects often available after transhepatic biopsy review. It is therefore enticing to suggest that systematic use of intraoperative frozen sections should be useful to avoid radical hepatectomy in order to decrease morbidity and mortality.

Hepatic endometrioma should always be considered in the differential diagnosis for a woman of any age presenting with a hepatic mass, with or without previous endometriosis history. The diagnosis is made on the basis of a histological examination, and a pericystectomy has been recommended.

## Conclusions

In summary, our 36-year-old Chinese primiparous female with hepatic endometriosis forming a large cystic mass was diagnosed with endometriosis through histological immunostaining without intrinsic abnormality. Our present case suggested that the liver maybe a transportation target of endometrial fragments by lymphatic or blood vessels, which provides a theoretical basis for the complex mechanisms of endometriosis.

## Consent

Written informed consent was obtained from the patient for publication of this case report and any accompanying images.
